# Clinical Features and Risk Factors for Lymph Node Metastasis in Early Signet Ring Cell Gastric Cancer

**DOI:** 10.3389/fonc.2021.630675

**Published:** 2021-07-09

**Authors:** Xiaoliang Jin, Wei Wu, Jing Zhao, Shuang Song, Chunli Zhang, Wenyong Sun, Bin Lv

**Affiliations:** ^1^ Department of Gastroenterology, First Affiliated Hospital of Zhejiang Chinese Medical University, Hangzhou, China; ^2^ Department of Pathology, Zhejiang Cancer Hospital, Hangzhou, China; ^3^ Department of Pathology, First Affiliated Hospital of Zhejiang Chinese Medical University, Hangzhou, China

**Keywords:** early gastric cancer, signet ring cell carcinoma, lymph node metastasis, clinical features, endoscopic submucosal dissection (ESD)

## Abstract

Signet ring cell carcinoma (SRCC) was previously thought to have a worse prognosis than differentiated gastric carcinoma (DC). However, recent studies have shown that its prognosis is related to staging. Here, we analyzed the clinicopathological features and the rate of lymph node metastasis (LNM) in 2166 patients with gastric cancer (605 early and 1561 advanced cases) who underwent gastrectomy and lymph node dissection (D2) from 2016 to 2019. The LNM rate for early and advanced cases was 18.0% and 74.2%, respectively. Regarding early cases, the LNM rate in SRCC was similar to that in DC (10% *vs.* 16.1%, *p*=0.224), and significantly lower than that in undifferentiated carcinoma (UDC; 10% *vs.* 23.3%, *p*=0.024). Tumor size, infiltration depth, pathological type, and mixed type were risk factors for LNM in early cases. Regarding intramucosal cases, the LNM rate in SRCC was similar to that in DC (4.3% *vs.* 3.7%, *p*=0.852), and significantly lower than that in UDC (11.2%). The LNM rate was significantly higher in submucosal than intramucosal cases (28.1% *vs.* 6.3%, *p*<0.001), and in early mixed cases than early pure cases (23.2% *vs.* 12.4%, *p*<0.001). Regarding early pure cases, the LNM rate in SRCC was similar to that in DC (9.3% *vs.* 7.2%, *p*=0.641), but significantly lower than that in UDC (9.3% *vs.* 24.7%, *p*=0.039). In summary, the LNM rate in early SRCC was similar to that in early DC but significantly lower than that in early UDC. Early SRCC fits with the endoscopic submucosal dissection (ESD) indication related to undifferentiated cases, and ESD may be effective. Additionally, the LNM rate was markedly higher for submucosal cases than intramucosal cases, and for mixed cases than pure cases.

## Introduction

Early gastric cancer refers to gastric cancer in which the tumor is limited to the mucosa and submucosa, regardless of lymph node metastasis (LNM). Endoscopic submucosal dissection (ESD) is a first-line treatment for early gastric cancer, enabling patients to avoid radical surgery, preserve organ integrity, and maintain a good quality of life (1). As ESD does not involve lymph node dissection, it is suitable for cases of early gastric cancer with almost no risk of LNM. LNM in early gastric cancer is mainly affected by tumor size, invasion depth, pathological type, and the presence of ulcers (2).

Signet ring cell carcinoma (SRCC) is a type of gastric cancer in which ≥50% of the tumor cells are signet ring cells (3). Laurén (4) classified it as a diffuse type. For a long time, SRCC was considered to be highly malignant with a poor prognosis (5, 6). However, this was mainly based on cases of advanced SRCC. In recent years, with the increased detection rate and deepened understanding of early and advanced gastric cancer, it was found that the biological behavior differed between early and advanced SRCC. Early SRCC has low invasiveness and a similar prognosis to early differentiated carcinoma (DC), and both early SRCC and DC have a superior prognosis compared to early undifferentiated carcinoma (UDC) (2, 7). However, there are gaps in the literature regarding the difference in the LNM rate between early SRCC and non-SRCC cases and whether early SRCC can be treated with ESD (8–10). For these reasons, we aimed to compare the LNM rate between early SRCC and non-SRCC cases, intramucosal and submucosal cases, and pure and mixed cases. Additionally, we aimed to analyze the risk factors for LNM in early cases, advanced cases, undifferentiated type (comprising poorly differentiated and mucinous carcinoma), and SRCC.

## Methods

The Ethics Review Committee of the First Affiliated Hospital of Zhejiang Chinese Medical University approved the study (2020-KL-085-01). The Ethics Review Committee waived the need for written informed consent as (1) the main risk in this study was loss of subjects’ anonymity, and the informed consent forms would contain the only identifiable information, and (2) the study did not involve biological specimens so the risk of biological leakage was minimal.

We included patients with gastric cancer who underwent radical gastrectomy and lymph node dissection (D2) or additional radical gastrectomy and lymph node dissection (D2) after non-curative dissection by ESD at the First Affiliated Hospital of Zhejiang University of Traditional Chinese Medicine and Zhejiang Cancer Hospital from 2016 to 2019. The non-curative dissection conditions of ESD: (1) not included in the expanded ESD indications listed in the Japanese gastric cancer treatment guidelines, (2) positive incisal margin, and (3) lymphatic/vascular invasion. The exclusion criteria were as follows: (1) gastric metastatic cancer; (2) two or more lesions in the stomach; (3) preoperative chemotherapy, radiotherapy, or targeted biological therapy; and (4) other rare types of gastric cancer.

The clinical and pathological data of the included patients were obtained. All specimens, including the resected stomach and regional lymph nodes, were histologically examined by three independent senior pathologists. The data included sex, age, tumor size [≤2 or >2 cm, based on the ESD indications in the Japanese gastric cancer treatment guidelines (11)], macroscopic type (I, II a–c, or III), infiltration depth [early cases were divided into intramucosal and submucosal types, based on the 8th edition of the American Joint Committee on Cancer (AJCC) tumor, node, metastasis (TNM) gastric cancer staging system], distant metastasis status (based on the AJCC TNM system), pathological type [DC, UDC, or SRCC, based on the Japanese gastric cancer classification (12)], pure/mixed type, and LNM status [LNM (+) or LNM (-)]. Regarding the pathological types, DC comprised well and moderately differentiated adenocarcinoma, while UDC comprised poorly differentiated and mucinous adenocarcinoma. The early cases of each pathological type were further divided into pure type (pure DC, pure UDC, and pure SRCC) or mixed type [mixed DC, mixed UDC, and mixed SRCC, the latter of which was defined as the presence of other differentiated cells in SRCC tumors (13)], according to the tumor cell composition and the Japanese gastric cancer classification (12). Additionally, early SRCC was split into the ESD indication and non-indication groups, according to the Japanese gastric cancer treatment guidelines (11). In early and advanced cases, we analyzed the associations of LNM with tumor size, infiltration depth, pathological type, and pure/mixed type. We also analyzed the LNM rate and other clinicopathological features in early SRCC in the ESD indication or non-indication groups.

Statistical analysis was conducted using SPSS v25.0 software. The continuous data are expressed as mean ± SD, and the categorical data are expressed as frequency (%). We used the chi-square test, Fisher’s exact test, and the Monte Carlo method to assess the categorical variables, and binary logistic regression was used for multivariate analysis, with *p<*0.05 indicating statistical significance.

## Results

### Patient Characteristics and LNM Rate

We obtained data on 2166 patients with gastric cancer (1495 males and 671 females), with a mean age of 62 ± 5.7 years. Of these patients, 605 had early cancer, with 109 (18.0%) cases of LNM, and 1561 had advanced cancer, with 1158 (74.2%) cases of LNM. There were 983 (45.4%), 1062 (49.0%), and 121 (5.6%) cases of DC, UDC, and SRCC, respectively. There were 1079 (49.8%) pure and 1087 (50.2%) mixed cases.

**Figure 1:**
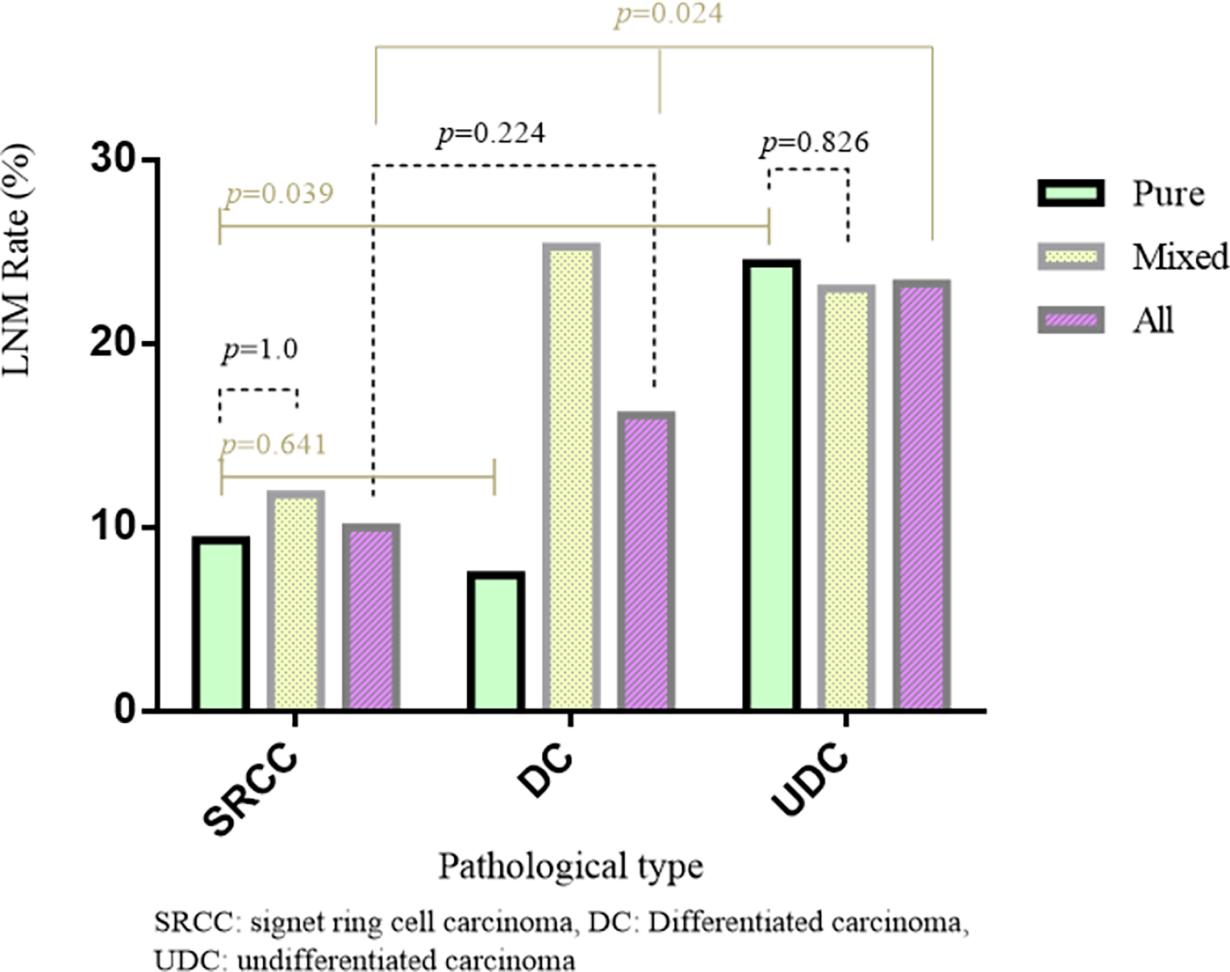
The difference of LNM between puremixed type in different pathological of EGC.

Regarding the early cases, the LNM rate in SRCC was non-significantly lower than that in DC (10.0% *vs.* 16.1%, *p*=0.224), but significantly lower than that in UDC (10% *vs.* 23.3%, p=0.024) (**Table 1** and **Figure 1**). Regarding the advanced cases, the LNM rate in SRCC was similar to that in UDC (82.0% *vs.* 79.0%, *p*=0.580), but significantly higher than that in DC (67.1%, *p*=0.017).

**Table 1:** Univariate analysis of the risk factors for lymph node metastasis in early and advanced gastric cancer.

Risk factor	Early (n = 605)			Advanced (n = 1561)		
	n	LNM (+)	*P*	n	LNM (+)	*P*
Sex					
Male	374	63 (16.8%)		1121	839 (74.8%)	
Female	231	46 (19.9%)	0.34	440	319 (72.5%)	0.341
Age (years)					
<65	411	75 (18.2%)		816	606 (74.3%)	
≥65	194	34 (17.5%)	0.829	745	552 (74.1%)	0.939
Tumor size (cm)					
≤2	392	47 (12.0%)		127	55 (43.3%)	
>2	213	62 (29.1%)	<0.001	1434	1103 (76.9%)	<0.001
T					
T1a	269	17 (6.3%)			
T1b	320	90 (28.1%)			
T2	–	–		289	127 (43.9%)	
T3	–	–		270	197 (73.0%)	
T4	–	–	<0.001	1002	834 (83.2%)	<0.001
Excluded	16	2 (12.5%)		–	–
M					
M0	–	–		1553	1151 (74.1%)	
M1	–	–		8	7 (87.5%)	0.688
Pathological type					
SRCC	60	6 (10%)		61	50 (82.0%)	
DC	335	54 (16.1%)		648	435 (67.1%)	
UDC	210	49 (23.3%)	0.024	852	673 (79.0%)	<0.001
Pure/mixed					
Pure	291	36 (12.4%)		788	570 (72.3%)	
Mixed	314	73 (23.2%)	<0.001	773	588 (76.1%)	0.092
Total	605	109 (18.0%)		1561	1158 (74.2%)	

DC, differentiated carcinoma; UDC, undifferentiated carcinoma; SRCC, signet ring cell carcinoma; P_Early SRCC vs. DC_=0.224, P_Early DC vs. UDC_=0.036, P_Early SRCC vs. UDC_=0.024, P_Advanced SRCC vs. DC_=0.017, P_Advanced DC vs. UDC_ <0.001, P_Advanced SRCC vs. UDC_=0.580.

The results of univariate analysis of the risk factors for LNM in early and advanced cases are shown in **Table 1**. The results of multivariate regression analysis suggested that tumor size, infiltration depth, pathological type, and mixed type were risk factors for LNM in early cases, while tumor size, infiltration depth, and pathological type were risk factors for LNM in advanced cases (**Table 2**).

**Table 2:** Multivariate analysis of the risk factors for lymph node metastasis in early and advanced gastric cancer.

Risk factor	Early			Advanced		
	*P*	OR	95% CI	*P*	OR	95% CI
Tumor size	<0.001	2.454	1.551-3.884	<0.001	2.612	1.749-3.902
T	<0.001	4.859	2.767-8.531	<0.001	1.877	1.672-2.108
Pathological type	0.026	1.586	1.057-2.380	0.010	1.318	1.066-1.631
Pure/mixed	0.003	2.030	1.270-3.246	–	–	–

### Difference in LNM Rate Between Submucosal and Intramucosal Cases

Among the 605 early cases, there were 269 intramucosal and 320 submucosal cases, after excluding 16 cases with incomplete records or unclear invasion depth (10 cases of DC, 6 cases of UDC). The clinicopathological features (including LNM status) of early DC, UDC, and SRCC are shown in **Table 3**, and those of intramucosal or submucosal DC, UDC, and SRCC are shown in **Table 4**. In intramucosal (*p*=0.079) or submucosal (*p*=0.329) cases, there was no significant difference in the LNM rate between the three different pathological types. However, the LNM rate was significantly higher in submucosal cases than intramucosal cases of the same pathological type (*p*=0.008, *p*<0.001, and *p*<0.001, respectively).

**Table 3:** Clinicopathological characteristics of early cases of three pathological types.

	DC (n = 335)	UDC (n = 210)	SRCC (n = 60)	*P*
Sex				
Male	235 (70.1%)	105 (50%)	34 (56.7%)	
Female	100 (29.9%)	105 (50%)	26 (43.3%)	<0.001
Age (years)				
<65	204 (60.9%)	161 (76.7%)	46 (76.7%)	
≥65	131 (39.1%)	49 (23.3%)	14 (23.3%)	<0.001
Tumor size (cm)				
≤2	198 (59.1%)	148 (70.5%)	46 (76.7%)	
>2	137 (40.9%)	62 (29.5%)	14 (23.3%)	0.003
Macroscopic type				
I	20 (6.0%)	0 (0%)	0 (0%)	
IIa	14 (4.2%)	6 (2.9%)	1 (1.7%)	
IIb	36 (10.7%)	20 (9.5%)	20 (33.3%)	
IIc	175 (52.2%)	123 (58.6%)	30 (50.0%)	
III	85 (25.4%)	61 (29.0%)	8 (13.3%)	
IIa+IIb	1 (0.3%)	0 (0%)	0 (0%)	
IIa+IIc	3 (0.9%)	0 (0%)	1 (1.7%)	
IIb+IIc	1 (0.3%)	0 (0%)	0 (0%)	<0.001
Pure/mixed				
Pure	167 (49.9%)	81 (38.6%)	43 (71.7%)	
Mixed	168 (50.1%)	129 (61.4%)	17 (28.3%)	<0.001
Infiltration depth				
Intramucosal	134 (40.0%)	89 (42.4%)	46 (76.7%)	
Submucosal	191 (57.0%)	115 (54.8%)	14 (23.3%)	<0.001
Excluded	10 (3.0%)	6 (2.8%)	0	
LNM				
+	54 (16.1%)	49 (23.3%)	6 (10%)	
-	281 (83.9%)	161 (76.7%)	54 (90%)	0.024

DC, differentiated carcinoma; UDC, undifferentiated carcinoma; SRCC, signet ring cell carcinoma.

**Table 4:** Lymph node metastasis in intramucosal and submucosal cases of three pathological types.

	DC (N = 335)			UDC (N = 210)			SRCC (N = 60)			*P*
	n	LNM (+)	*P*	n	LNM (+)	*P*	n	LNM (+)	*P*	
Intramucosal	134	5 (3.7%)		89	10 (11.2%)		46	2 (4.3%)		0.079
Submucosal	191	48 (25.1%)	0.008	115	38 (33.0%)	<0.001	14	4 (28.6%)	<0.001	0.329
Excluded	10	1 (10.0%)		6	1 (16.7%)		0	–		

DC, differentiated carcinoma; UDC, undifferentiated carcinoma; SRCC, signet ring cell carcinoma.

### Difference in LNM Rate Between Pure and Mixed Cases

Among the 605 early cases, there were 291 pure and 314 mixed cases. The LNM rate was significantly higher in mixed cases than pure cases (23.2% *vs.* 12.4%, *p*=0.001), and significantly higher in mixed DC than pure DC (25.0% *vs.* 7.2%, *p*<0.001). However, in early UDC or SRCC, the difference in LNM rate between mixed and pure cases was not significant. The LNM rate was similar in pure SRCC and pure DC (9.3% *vs.* 7.2%, *p*=0.641), and significantly lower in pure SRCC than pure UDC (9.3% *vs.* 24.7%, *p*=0.039) (**Figure 1**).The LNM rate was significantly higher in mixed cases than pure cases for both intramucosal (9.9% *vs.* 2.9%, *p*=0.018) and submucosal (33.9% *vs.* 21.2%, *p*=0.012) cases (**Table 5**) (after excluding 16 cases with unclear invasion depth).

**Table 5:** Lymph node metastasis in pure and mixed early gastric cancer.

	Pure (n = 291)		Mixed (n = 314)		*P*
	n	LNM+ (%)	n	LNM+ (%)	
Pathological type					
SRCC	43	4 (9.3% )	17	2 (11.8%)	1.0
DC	167	12 (7.2% )	168	42 (25%)	<0.001
UDC	81	20 (24.7%)	129	29 (22.5%)	0.712
All	291	36 (12.4%)	314	73 (23.2%)	0.001
Infiltration depth					
Intramucosal	138	4 (2.9%)	131	13 (9.9%)	0.018
Submucosal	146	31 (21.2%)	174	59 (33.9%)	0.012
Excluded	7	1 (14.3%)	9	1 (11.1%)	1.0

DC, differentiated carcinoma; UDC, undifferentiated carcinoma; SRCC, signet ring cell carcinoma.

### Difference in LNM Rate in Early SRCC, Poorly Differentiated Carcinoma, and Mucinous Carcinoma

Among the 605 early cases, 270 cases were undifferentiated type, comprising 60 cases of SRCC (which is considered a subtype of the undifferentiated type), 204 cases of poorly differentiated carcinoma, and 6 cases of mucinous carcinoma. To determine the risk factors for LNM, we conducted univariate and multivariate analyses. The results suggested that tumor size and infiltration level (submucosal) were independent risk factors for LNM (**Table 6**). The LNM rate in early SRCC was 10%, which was significantly lower than that in early poorly differentiated carcinoma (24%, *p*=0.019).

**Table 6:** Univariate and multivariate analyses of the risk factors for lymph node metastasis in early undifferentiated gastric cancer.

Risk factor	Univariate analysis (n = 270)			Multivariate analysis (n = 270)		
	n	LNM (+)	*P*	*P*	OR	95%CI
Sex						
Male	139	25 (18.0%)				
Female	131	30 (22.9%)	0.316			
Age (years)						
<65	207	44 (21.3%)				
≥65	63	11 (17.5%)	0.512			
Tumor size (cm)				0.005	2.519	1.313-4.829
≤2	194	28 (14.4%)				
>2	76	27 (35.5%)	<0.001			
Infiltration depth				<0.001	3.993	1.920-8.305
Intramucosal	135	12 (8.9%)				
Submucosal	129	42 (32.6%)	<0.001			
Excluded	6	1 (16.7%)				
Pathological type				0.617	1.225	0.553-2.712
SRCC	60	6 (10.0%)				
PDC	204	49 (24.0%)				
MGC	6	0 (0%)	0.027			
Pure/mixed						
Pure	124	24 (19.4%)				
Mixed	146	31 (21.2%)	0.703			
Total	270	55 (20.4%)				

MGC, mucinous carcinoma; PDC, poorly differentiated carcinoma; SRCC, signet ring cell carcinoma.

### Clinicopathological Features of SRCC in the ESD Indication and Non-Indication Groups

Among the 60 cases of early SRCC, there were 38 cases in the ESD indication group. The clinicopathological features of SRCC in the ESD indication and non-indication groups are shown in **Table 7**. Further analysis showed that 2 out of the 38 cases (5.3%) of SRCC and 8 out of the 66 cases (12.1%) of undifferentiated type (i.e., poorly differentiated and mucinous carcinoma) in the ESD indication group exhibited LNM, and the clinicopathological features of these cases are shown in **Table 8**.

**Table 7:** Clinicopathological characteristics of SRCC according to ESD indication.

	Yes (n = 38)	No (n = 22)	*P*
Sex			
Male	23 (60.5%)	11 (50%)	
Female	15 (39.5%)	11 (50%)	0.428
Age (years)			
<65	31 (81.6%)	15 (68.2%)	
≥65	7 (18.4%)	7 (31.8%)	0.237
Macroscopic type			
IIa	1 (2.6%)	0 (0%)	
IIb	16 (42.1%)	4 (18.2%)	
IIc	20 (52.6%)	10 (45.5%)	
III	0 (0%)	8 (36.4%)	
IIa+IIc	1 (2.6%)	0 (0%)	<0.001
Pure/mixed			
Pure	31 (81.6%)	12 (54.5%)	
Mixed	7 (18.4%)	10 (45.5%)	0.025
LNM			
(+)	2 (5.3%)	4 (18.2%)	
(-)	36 (94.7%)	18 (81.8%)	0.179

ESD, endoscopic submucosal dissection; SRCC, signet ring cell carcinoma.

**Table 8:** Pathological characteristics of cancers with ESD indication but with lymph node metastasis.

Pathological type	Age	Sex	Size	Infiltration depth	Pure/mixed	Macroscopic type
SRCC	54	F	0.9×0.5×0.3	Intramucosal	Pure	IIc
SRCC	66	F	1.5×1×0.5	Intramucosal	Pure	IIb
PDC	64	M	2×0.8×0.2	Intramucosal	Mixed	IIb
PDC	50	M	0.8×0.7×0.4	Intramucosal	Pure	IIc
PDC	58	F	1.2×1.0×0.3	Intramucosal	Mixed	IIc
PDC	61	F	1.8×0.6×0.2	Intramucosal	Mixed	IIc
PDC	54	M	1.0×1.0×0.2	Intramucosal	Mixed	IIc
PDC	57	M	0.9×0.9×0.3	Intramucosal	Mixed	IIc
PDC	58	F	1.5×1.0×0.2	Intramucosal	Mixed	IIc
PDC	64	M	2.0×2.0×0.3	Intramucosal	Mixed	IIc

ESD, endoscopic submucosal dissection; PDC, poorly differentiated carcinoma; SRCC, signet ring cell carcinoma.

## Discussion

As the fifth most common cancer in the world, gastric cancer is a significant threat to human health. Recently, its incidence (mainly the intestinal type) in Asia has declined, which may be related to the gradually increasing focus on and treatment of *Helicobacter pylori* in Asia (14). However, the incidence of SRCC is rising, which necessitates more attention. The biological behavior of a case is very important when assessing whether ESD is feasible or not. Our study showed that the LNM rate in early SRCC was slightly but non-significantly lower than that in early DC, but significantly lower than that in early UDC (i.e., poorly differentiated and mucinous carcinoma). Additionally, early mixed cases had a higher LNM rate than early pure cases. Regarding the biological behavior of LNM, our study verified the feasibility of using ESD for early SRCC.

SRCC usually occurs in young women and is related to estrogen. Yang et al. (15) reported that >80% of SRCC cases could produce and secrete mucin and expressed estrogen receptors, which affected tumor growth and invasion, and the undifferentiated type is considered an independent risk factor for LNM among early cases. SRCC, as a subtype of the undifferentiated type, was previously considered to have increased malignant behavior, poor prognosis, and a high risk of LNM (5, 6). Compared to other gastric cancers, E-cadherin is downregulated in SRCC, which decreases cell adhesion and increases invasion of neighboring tissues (16–18). However, recent studies have found that the prognosis and biological behavior differ between early and advanced SRCC (7, 19).

Hyung et al. (7) studied 933 early gastric cancer cases and found that the LNM rate in early SRCC was significantly lower than that in early non-SRCC cases (5.9% *vs.* 16.0%, *p*<0.001), and multivariate analysis of early cases showed that SRCC was an independent protective factor against LNM, the 10-year survival rate was significantly better for early SRCC than early non-SRCC cases (89.7% *vs.* 79.1%, *p*=0.01). However, a large (n=2971) study by Kao et al. (2) showed that the LNM rate in early SRCC was similar to that in early non-SRCC cases (15.7% *vs.* 13.4%, *p*=0.433), despite this, the 5-year overall and disease-free survival rates were considerably higher for SRCC than non-SRCC cases (90.7% *vs.* 83.2%, *p*=0.001; 87.4% *vs* 81.6%, *p*=0.003 respectively). We found that the LNM rate in early SRCC was slightly but non-significantly lower than that in early DC, and both LNM rates were significantly lower than that in early UDC (*p*=0.024 and *p*=0.036, respectively). This shows that the LNM rate in early SRCC is similar to the early DC; notably, the absolute indication for ESD is DC without ulcerative findings (UL0) and with an invasion depth clinically diagnosed as T1a and a diameter ≤2 cm. Our other result was that, in advanced cases, the LNM rate in SRCC was similar to that in UDC, and both LNM rates were significantly higher than that in DC.

According to the invasion depth, early cases can be divided into intramucosal and submucosal types. Previous research showed that the LNM rates in intramucosal and submucosal carcinoma were around 3.2% (0.0–20.3%) and 19.2% (10.2–33.0%), respectively (20, 21). In our study, the LNM rates of intramucosal and submucosal SRCC were 4.3% and 28.6%, respectively. The LNM rates of intramucosal and submucosal DCs were 3.7% and 25.1%, respectively, consistent with previous research. However, the LNM rates of intramucosal and submucosal UDC were 11.2% and 33.0%, respectively, which is higher than in previous research (4.2% and 19.0%, respectively) (22, 23). We analyzed the clinicopathological data of intramucosal and submucosal UDC further, and we found that the rates of mixed cases in intramucosal and submucosal UDC were 68.5% (61/89) and 56.5% (65/115), respectively. Additionally, according to multivariate analysis, mixed type was a risk factor for LNM in early cases. This may explain the differences in the LNM rates in intramucosal and submucosal UDC between our study and the previous research. Our data also showed that among intramucosal cases, the LNM rate in SRCC was similar to that in DC (4.3% and 3.7%, respectively), but significantly lower than that in UDC (11.2%). Still, the LNM rate increased if the cancer invaded the submucosa, so ESD is not suitable for submucosal undifferentiated cases, including submucosal SRCC.

In various pathological types of gastric cancer, having the mixed type increases the LNM rate and worsens the prognosis. Huh et al. (24) showed that the LNM rate was higher in early mixed SRCC than in early pure SRCC (19.2% *vs.* 5.9%, *p*<0.001), and mixed SRCC was an independent risk factor for LNM in early cases (OR=2.30, *p*=0.001). Hu et al. (12) also showed that mixed SRCC was more aggressive than pure SRCC. We found that, among early cases, the LNM rate was significantly higher in the mixed type than the pure type (23.2% *vs.* 12.4%, *p*=0.001). Additionally, in intramucosal cases, submucosal cases, and DC, the LNM rate was higher in the mixed type than pure type (*p*=0.018, *p*=0.012, *p*<0.001, respectively). However, in UDC, there was no significant difference between the mixed and pure types. Reviewing the raw data on early UDC with LNM, we found that pure UDC tended to be larger (>2cm: 55%) and deeper (submucosa: 90%) and had more ulcers (type III: 55%) than mixed UDC (>2 cm: 44.8%; submucosa: 69.0%; type III: 41.4%), and the tumor size, invasion depth, and presence of ulcers were all risk factors for LNM in early cases. In early SRCC, the LNM rate was lower in the pure type than the mixed type (9.3% *vs* 11.8%), but not significantly, which may be attributable to the small sample size.

We further analyzed the LNM in three undifferentiated types (early SRCC, poorly differentiated carcinoma, and mucinous carcinoma). The results suggested that tumor size and infiltration level were independent risk factors for LNM. The pathological type had a significant effect in univariate analysis, but not in the multivariate analysis (**Table 6**). The sample size (i.e., the small number of cases of mucinous carcinoma) may have influenced the result, so we analyzed the difference between the SRCC and poorly differentiated carcinoma. The former had a significantly lower LNM rate. This indicates that SRCC is a pathological type with a lower LNM rate. In the future, SRCC should be compared to mucinous carcinoma using a larger sample.

In the most recent multicenter study in Japan, Takizawa et al. analyzed 275 cases of early undifferentiated gastric cancer treated with ESD. They showed that 71% of the patients were cured, with a recurrence rate of 0% during the 5-year follow-up, while the success rate of surgery after non-curative ESD was 98.9%. The 5-year overall and recurrence-free survival rates were 99.3% and 98.9%, respectively, indicating the efficacy and safety of ESD for early undifferentiated gastric cancer (25). Lee et al. (9) proposed that endoscopic treatment was more suitable for early SRCC than for moderately and poorly differentiated types. We found that the LNM rate in SRCC in the ESD indication group was 5.3%, which is lower than the 11.9% reported by Zhu et al. (10). Furthermore, in the ESD indication group, 8 (12.1%) cases with poorly differentiated type had LNM, while only 2 (5.3%) cases with SRCC had LNM (**Table 8**). This suggests that treating SRCC with ESD is safer than treating UDC with it. Unfortunately, the clinicopathological characteristics of cases of SRCC with LNM in the ESD indication group could not be analyzed further because of the small sample size. However, regarding the cases of poorly differentiated carcinoma in the ESD indication group, more attention should be paid to LNM during follow-up after ESD when the macroscopic type is IIc and the cancer is the mixed type.

According to the above results, we believe that early SRCC is a special type of undifferentiated gastric cancer. The LNM rate was consistent with that in early DC, and so early SRCC with ESD indication can be treated endoscopically.

There are two major limitations in our study. First, as a retrospective study, there may have been selection bias, and the clinical and pathological data of some patients are incomplete. Second, follow-up data on the surgical patients were not obtained, so we did not perform a survival analysis; thus, inferences related to prognosis need to be made with caution.

## Conclusion

The LNM rate in early SRCC is similar to that in early DC, but significantly lower than that in early UDC. Early SRCC fits with the expanded ESD indication related to undifferentiated cases and so ESD may be an effective treatment, indicating that early SRCC is generally less dangerous than early UDC. The LNM rate is significantly higher in submucosal than intramucosal cases, and in early mixed cases than early pure cases. Tumor size, infiltration depth, pathological type, and mixed type are risk factors for LNM in early cases.

## Data Availability Statement

The original contributions presented in the study are included in the article/supplementary material, further inquiries can be directed to the corresponding author.

## Ethics Statement

The studies involving human participants were reviewed and approved by the Ethics Review Committee of the First Affiliated Hospital of Zhejiang Chinese Medical University. Written informed consent for participation was not required for this study in accordance with the national legislation and the institutional requirements.

## Author Contributions

XJ, WW, SS, CZ, and WS collected and analyzed the data. XJ, BL, and JZ designed the research methods. XJ and BL wrote the draft of the article. All authors contributed to the article and approved the submitted version.

## Funding

This study was supported by the Natural Science Foundation of China (NSFC) (Grant numbers 81970470 and 81770535), and Collaboration of Chinese traditional and Modern Medicine in Gastric Cancer.

## Conflict of Interest

The authors declare that the research was conducted in the absence of any commercial or financial relationships that could be construed as a potential conflict of interest.
